# Pilot Evaluation of the Long-Term Reproducibility of Capillary Zone Electrophoresis–Tandem Mass Spectrometry for Top-Down Proteomics of a Complex Proteome Sample

**DOI:** 10.1021/acs.jproteome.3c00872

**Published:** 2024-02-28

**Authors:** Seyed Amirhossein Sadeghi, Wenrong Chen, Qianyi Wang, Qianjie Wang, Fei Fang, Xiaowen Liu, Liangliang Sun

**Affiliations:** † Department of Chemistry, Michigan State University, 578 S Shaw Lane, East Lansing, Michigan 48824, United States; ‡ Department of BioHealth Informatics, Indiana University-Purdue University Indianapolis, 535 W Michigan Street, Indianapolis, Indiana 46202, United States; § Deming Department of Medicine, School of Medicine, Tulane University, 1441 Canal Street, New Orleans, Louisiana 70112, United States

**Keywords:** top-down proteomics, capillary zone electrophoresis, mass spectrometry, proteoform, reproducibility, label-free quantification, yeast cell lysate

## Abstract

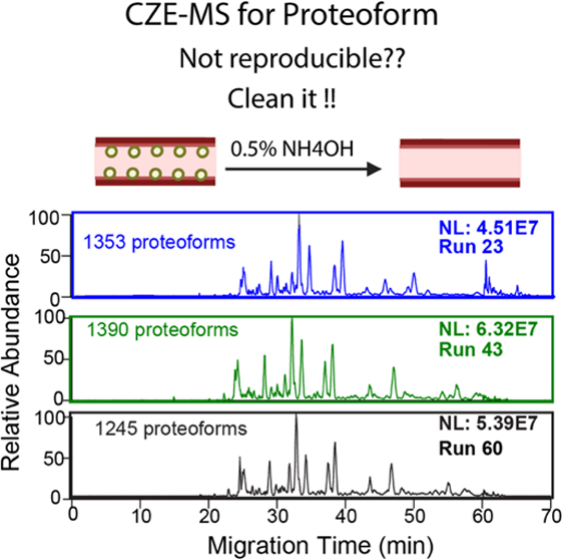

Mass spectrometry (MS)-based top-down proteomics (TDP) has revolutionized biological research by measuring intact proteoforms in cells, tissues, and biofluids. Capillary zone electrophoresis–tandem MS (CZE-MS/MS) is a valuable technique for TDP, offering a high peak capacity and sensitivity for proteoform separation and detection. However, the long-term reproducibility of CZE-MS/MS in TDP remains unstudied, which is a crucial aspect for large-scale studies. This work investigated the long-term qualitative and quantitative reproducibility of CZE-MS/MS for TDP for the first time, focusing on a yeast cell lysate. Over 1000 proteoforms were identified per run across 62 runs using one linear polyacrylamide (LPA)-coated separation capillary, highlighting the robustness of the CZE-MS/MS technique. However, substantial decreases in proteoform intensity and identification were observed after some initial runs due to proteoform adsorption onto the capillary inner wall. To address this issue, we developed an efficient capillary cleanup procedure using diluted ammonium hydroxide, achieving high qualitative and quantitative reproducibility for the yeast sample across at least 23 runs. The data underscore the capability of CZE-MS/MS for large-scale quantitative TDP of complex samples, signaling its readiness for deployment in broad biological applications. The MS RAW files were deposited in ProteomeXchange Consortium with the data set identifier of PXD046651.

## Introduction

Mass spectrometry (MS)-based top-down proteomics (TDP) is a powerful technique for the identification and quantification of proteoforms in biological samples.^1^ During the last several years, TDP has been deployed widely to discover new proteoform biomarkers of various diseases, e.g., cancer,^2−5^ neurodegeneration,^6−9^ cardiovascular diseases,^10^ infectious disease,^11−14^ and immunobiology.^15^ MS-based TDP is providing more and more new insights into the functions of proteins in modulating cellular processes.

Due to the high complexity of the proteoforms in cells or tissues, high peak capacity separation of proteoforms before MS is crucial. Liquid chromatography (LC)-MS has been the widely used technique for TDP of complex samples.^16,17^ Capillary zone electrophoresis (CZE) offers highly efficient separations of biomolecules according to electrophoretic mobility (μ_ef_), which relates to their charge-to-size ratios.^18^ CZE-MS has also been well recognized as an alternative technique to LC-MS for global TDP profiling of proteoforms in cells and tissues due to its high efficiency and sensitivity for proteoform separation and detection as well as its unique opportunity for accurate prediction of proteoform’s μ_ef_.^19−21^ Several research groups have shown the early examples of CZE-MS for highly sensitive and global TDP of complex biological samples.^22−25^ Our group has shown the identification of hundreds to thousands of proteoforms from complex samples by single-shot CZE-MS measurements via innovations in capillary coating, online proteoform stacking, etc.^19,20,26^ We further boosted the number of identified proteoforms from human cell lines to over 23,000 by coupling LC fractionation to CZE-MS.^3^ Most recently, we developed online two-dimensional high-field asymmetric waveform ion mobility spectrometry-CZE-MS (FAIMS-CZE-MS) to benefit the identification of large proteoforms^27^ and histone proteoforms.^28^ We also showed the capability of CZE-MS for TDP of membrane proteins.^29^ The Kelleher group documented the high sensitivity of CZE-MS for TDP and the reasonable complementarity between CZE-MS and LC-MS for proteoform identification.^30^ The Ivanov group illustrated the potential of CZE-MS for TDP of single mammalian cells.^31^

CZE-MS has made drastic progress in TDP and has been widely accepted as a useful tool for proteoform characterization. However, to use CZE-MS for large-scale TDP studies, we need to validate its long-term reproducibility for the top-down MS measurement of complex samples. In this work, for the first time, we performed a pilot investigation of the long-term reproducibility of CZE-MS for TDP of a complex sample (i.e., a yeast cell lysate) to achieve a better understanding of advantages, issues, and potential solutions of CZE-MS for large-scale TDP.

## Experimental Section

### Chemicals and Materials

Ammonium bicarbonate (ABC), ammonium hydroxide (NH_4_OH), 3-(trimethoxysilyl) propyl methacrylate, and Amicon Ultra (0.5 mL, 10 kDa cutoff size) centrifugal filter units and (Yeast Extract–Peptone–Dextrose) YPD Broth were ordered from Sigma-Aldrich (St. Louis, MO). LC/MS-grade water, acetonitrile (ACN), HPLC-grade acetic acid (AA), and fused silica capillaries (50 mm i.d., 360 mm o.d., Polymicro Technologies) were purchased from Fisher Scientific (Pittsburgh, PA). Acrylamide was obtained from Acros Organics (Fair Lawn, NJ). Complete, mini protease inhibitor cocktail (provided in EASYpacks) was purchased from Roche (Indianapolis, IN).

### Sample Preparation

Yeast growth in (Yeast Extract–Peptone–Dextrose) YPD Broth is meticulously cultivated using a well-defined procedure. To begin, 50 g of YPD Broth was blended with 1 L of distilled water, ensuring a precise mixture. This suspension underwent autoclaving at 121 °C for a duration of 15 min. Following this, yeast cultures are introduced into detergent-free containers. A brief vortexing was then carried out to uniformly disperse the yeast cells throughout the medium. The yeast cultures were subsequently nurtured in a shaking incubator at 300 rpm.

After yeast cell collection and cleanup with a PBS, 5 g of yeast cells was suspended in the lysis buffer containing 8 M urea, complete protease inhibitors and PhosSTOP (Roche), and 100 mM ammonium bicarbonate (pH 8.0), followed by incubation on ice for 30 min with periodical vortexing. The cells were lysed for 3 min using a homogenizer (Fisher Scientific) and then sonicated under a 50% duty cycle, level 10 output for 20 min on ice with a Branson Sonifier 250 (VWR Scientific). The yeast lysate was centrifuged at 14,000*g* for 10 min at 4 °C to collect the supernatant containing extracted proteins. The concentration of total proteins was measured by a bicinchoninic acid (BCA) kit (Fisher Scientific) according to the manufacturer’s instructions, and the sample was stored at −80 °C.

### Buffer Exchange

In this study, an Amicon Ultra Centrifugal Filter (Sigma-Aldrich) with a molecular weight cutoff (MWCO) of 10 kDa was utilized for buffer exchange to eliminate the urea effectively from protein samples. The procedure began with the initial wetting of the filter using 20 μL of 100 mM ammonium bicarbonate, followed by centrifugation at 14,000*g* for 10 min. Subsequently, an aliquot of 200 μg of proteins was added to the filter, and centrifugation was carried out for 20 min at 14,000*g*. 200 μL of 100 mM ammonium bicarbonate was added to the filter, followed by centrifugation at 14,000*g* for 20 min. This step was repeated twice to remove the urea and other small interferences completely. The final protein solution in 35 μL of 100 mM ammonium bicarbonate (protein concentration of 3 mg/mL) was collected for CZE-MS analysis. All centrifugation steps were performed at 4 °C.

### Preparation of Linear Polyacrylamide (LPA)-Coated Capillary

An LPA-coated capillary (1 m, 50 μm i.d., 360 μm o.d.) was prepared according to our previous procedure with minor modifications.^32^ First, 3 μL of ammonium persulfate (APS) solution (5% [w/v] in water) was added to 500 μL of acrylamide solution (4% [w/v] in water), and the mixture was degassed with nitrogen gas for 5 min to remove the oxygen in the solution. Then, the mixture was loaded into the pretreated capillary using a vacuum, followed by sealing both ends of the capillary with silica rubber and incubating it in a water bath at 50 °C for 40 min. Finally, a small portion (∼5 mm) of the capillary from both ends was removed with a cleaving stone, and the unreacted solution (an agarose gel-like consistency) was pushed out of the capillary with water (200 μL), using the syringe pump. One end of the separation capillary was etched by hydrofluoric acid to reduce its outer diameter to around 100 μm.^33^

### CZE-ESI-MS/MS Analysis

The automated CE operation was performed using an ECE-001 CE autosampler from CMP Scientific (Brooklyn, NY). Through an electro-kinetically pumped sheath flow CE-MS interface (CMP Scientific, Brooklyn, NY), the CE system was coupled to a Q-Exactive HF mass spectrometer (Thermo Fisher Scientific).^34,35^ For CZE separation, the LPA-coated capillary (50 μm i.d., 360 μm o.d., 1 m in length) was used. A background electrolyte (BGE) of 5% (v/v) acetic acid (pH 2.4) was used for CZE. The sample buffer was 100 mM ammonium bicarbonate (pH 8). The dramatic difference of BGE and sample buffer in pH enabled online dynamic pH junction-based sample stacking.^26^ The sheath buffer contained 0.2% (v/v) formic acid and 10% (v/v) methanol. The sample was injected into the capillary by applying pressure. The sample injection volume was calculated based on the pressure and injection time using Poiseuille’s law. In this study, 5 psi for a 20 s period was applied for sample injection, corresponding to about 100 nL of sample-loading volume for a 1 m long separation capillary (50 μm i.d.). At the injection end of the separation capillary, a high voltage (30 kV) was applied for separation, and in the sheath buffer vial, a voltage of 2–2.2 kV was applied for ESI. With a Sutter P-1000 flaming/brown micropipet puller, ESI emitters were pulled from borosilicate glass capillaries (1.0 mm o.d., 0.75 mm i.d., and 10 cm length). ESI emitters had an opening size of 25–35 μm.

All experiments were conducted using a Q-Exactive HF mass spectrometer. A data-dependent acquisition (DDA) method was used for the yeast protein sample. MS parameters were 120,000 mass resolution (at *m*/*z* 200), three microscans, 3E6 AGC target value, 100 ms maximum injection time, and 600–2000 *m*/*z* scan range. For MS/MS, 60,000 mass resolution (at 200 *m*/*z*), 1 microscan, 1E6 AGC, 200 ms injection time, 4 *m*/*z* isolation window, and 20% normalized collision energy (NCE) were used. The top 8 most intense precursor ions in one MS spectrum were isolated in the quadrupole and fragmented via higher-energy collision dissociation (HCD). Fragmentation was performed only on ions with intensities greater than 1E4 and charge states greater than 5. We enabled dynamic exclusion with a duration of 30 s. The “Exclude isotopes” function was enabled.

### Data Analysis

The complex sample data was analyzed using Xcalibur software (Thermo Fisher Scientific) to get the intensity and migration time of proteins. For the final figures, the electropherograms were exported from Xcalibur and formatted using Adobe Illustrator.

Proteoform identification and quantification were performed on the yeast protein RAW files using the TopPIC (Top-down mass spectrometry-based Proteoform Identification and Characterization) pipeline.^36^ In the first step, RAW files were converted into mzML files using the Msconvert tool.^37^ The spectral deconvolution which converted precursor and fragment isotope clusters into the monoisotopic masses and proteoform features were then performed using TopFD (Top-down mass spectrometry Feature Detection, version 1.5.6).^38^ The resulting mass spectra and proteoform feature information were stored in msalign and text files, respectively. The database search was performed using TopPIC (version 1.5.6) against UniProt proteome database of Yeast (UP000002311, 6060 entries, version 11/14/2022) concatenated with a shuffled decoy database of the same size as the yeast database. The maximum number of unexpected mass shifts was one. The mass error tolerances for precursors and fragments were 15 parts per million (ppm). There was a maximum mass shift of 500 Da for the unknown mass shifts. To estimate false discovery rates (FDRs) of proteoform identifications, the target-decoy approach was used and proteoform identifications were filtered by a 1% FDR at the proteoform-spectrum-match (PrSM) level and proteoform level.^39,40^ The lists of identified proteoforms from all CZE-MS/MS runs are shown in Supporting Information I. The TopDiff (Top-down mass spectrometry-based identification of Differentially expressed proteoforms, version 1.5.6) software was used to perform label-free quantification of identified proteoforms by CZE-MS/MS using default settings.^41^ The MS RAW files were deposited to the ProteomeXchange Consortium via the PRIDE^42^ partner repository with the data set identifier of PXD046651.

### Capillary Cleanup

To remove proteins adsorbed on the capillary inner wall, the capillary was cleaned periodically by flushing with 0.5% NH_4_OH for 10 min at 30 psi, H_2_O for 10 min at 20 psi, and the BGE (5% acetic acid) for 10 min at 20 psi successively.

## Results and Discussion

**Figure 1:**
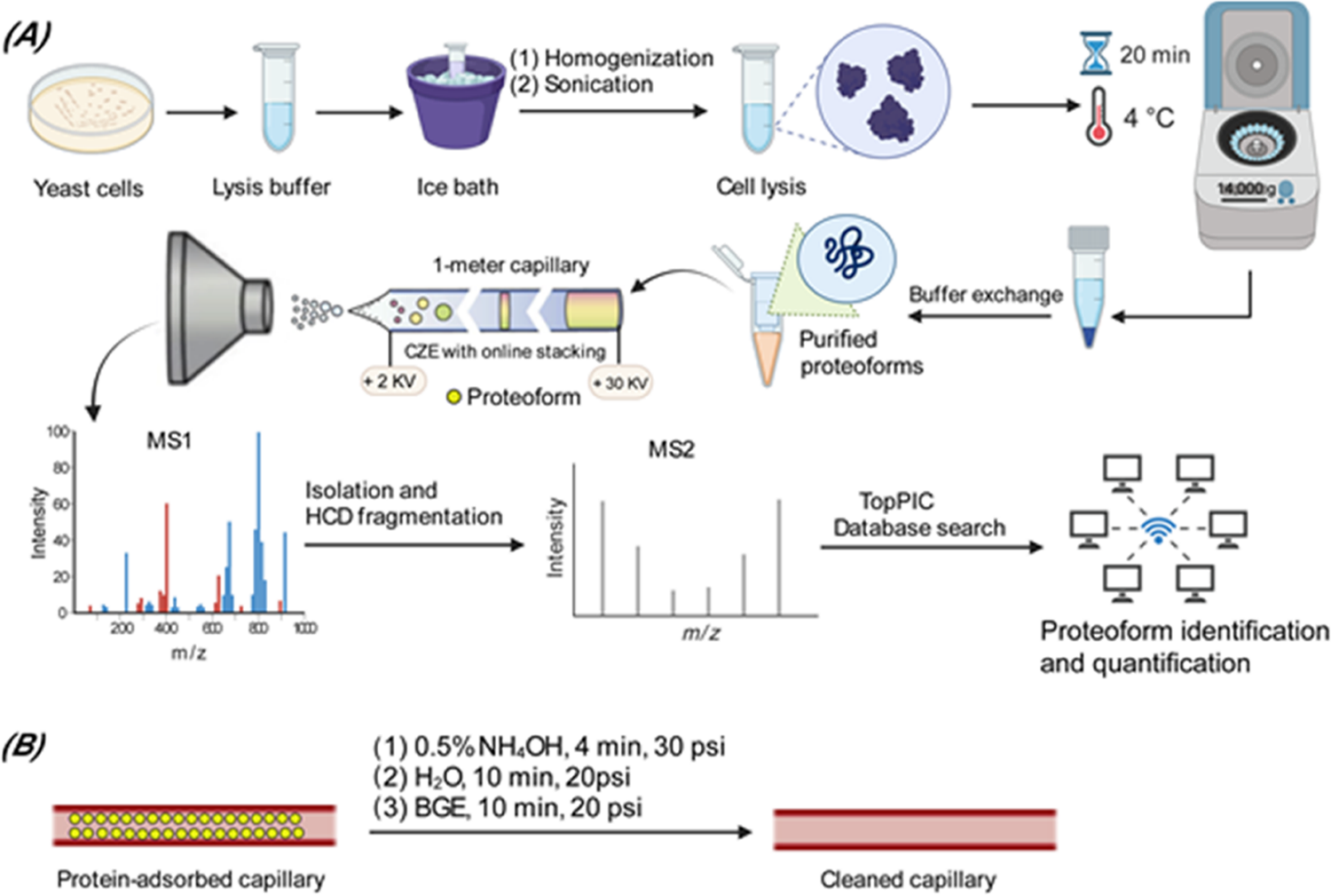
(A) Schematic of the experimental design of sample preparation, CZE-MS/MS analysis, and database search. (B) Schematic of the capillary inner-wall cleanup procedure using NH_4_OH. The figure is created using the BioRender and is used here with permission.

For the first time, we studied the long-term reproducibility of CZE-MS/MS for TDP of a complex proteome sample, a yeast cell lysate, and developed an effective procedure for cleaning up the inner wall of LPA-coated capillaries for reproducible CZE-MS/MS measurements of proteoforms. Figure 1A shows the experimental design of this project. Yeast cells were lysed by homogenization and sonication. The proteoform extract was analyzed by the dynamic pH junction-based CZE-MS/MS^26^ after a simple buffer exchange with a 10 kDa cutoff centrifugal filter unit. The yeast cell lysate was diluted to 1 mg/mL with 100 mM ammonium bicarbonate (pH 8) for CZE-MS/MS. Finally, the TopPIC software developed by Liu’s group was used for database search to identify and quantify proteoforms. Figure 1B represents the cleanup procedure to remove the adsorbed proteoforms on the LPA polymer coating on the capillary inner wall.

### Reproducibility of CZE-MS/MS for Top-Down Proteomics of a Complex Sample

**Figure 2:**
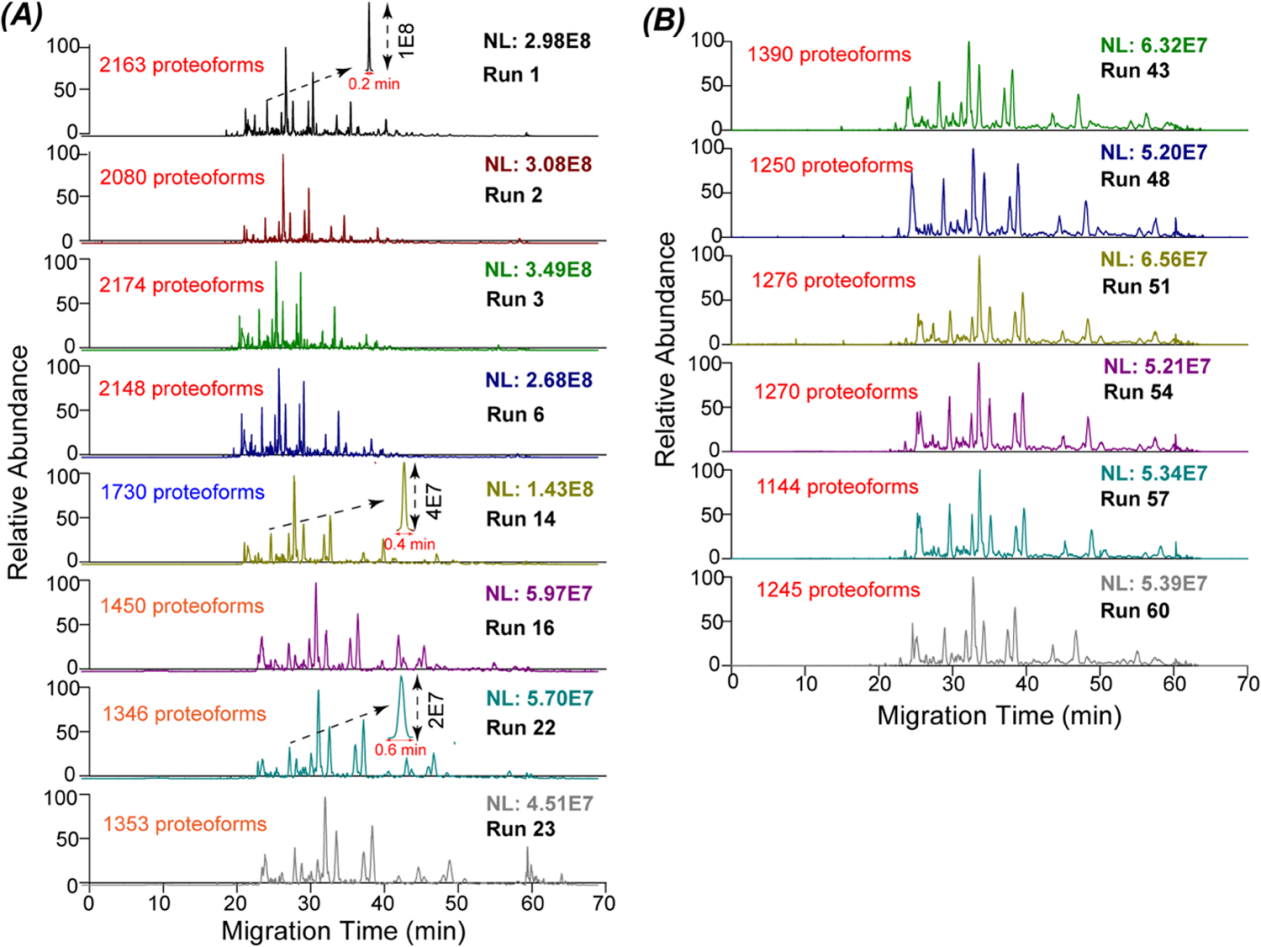
Electropherograms of a yeast cell lysate after analysis by CZE-MS/MS. (A) Example runs during the first 23 CZE-MS/MS measurements. (B) Six examples of CZE-MS/MS runs during the 40th to 62nd measurements.

**Figure 3:**
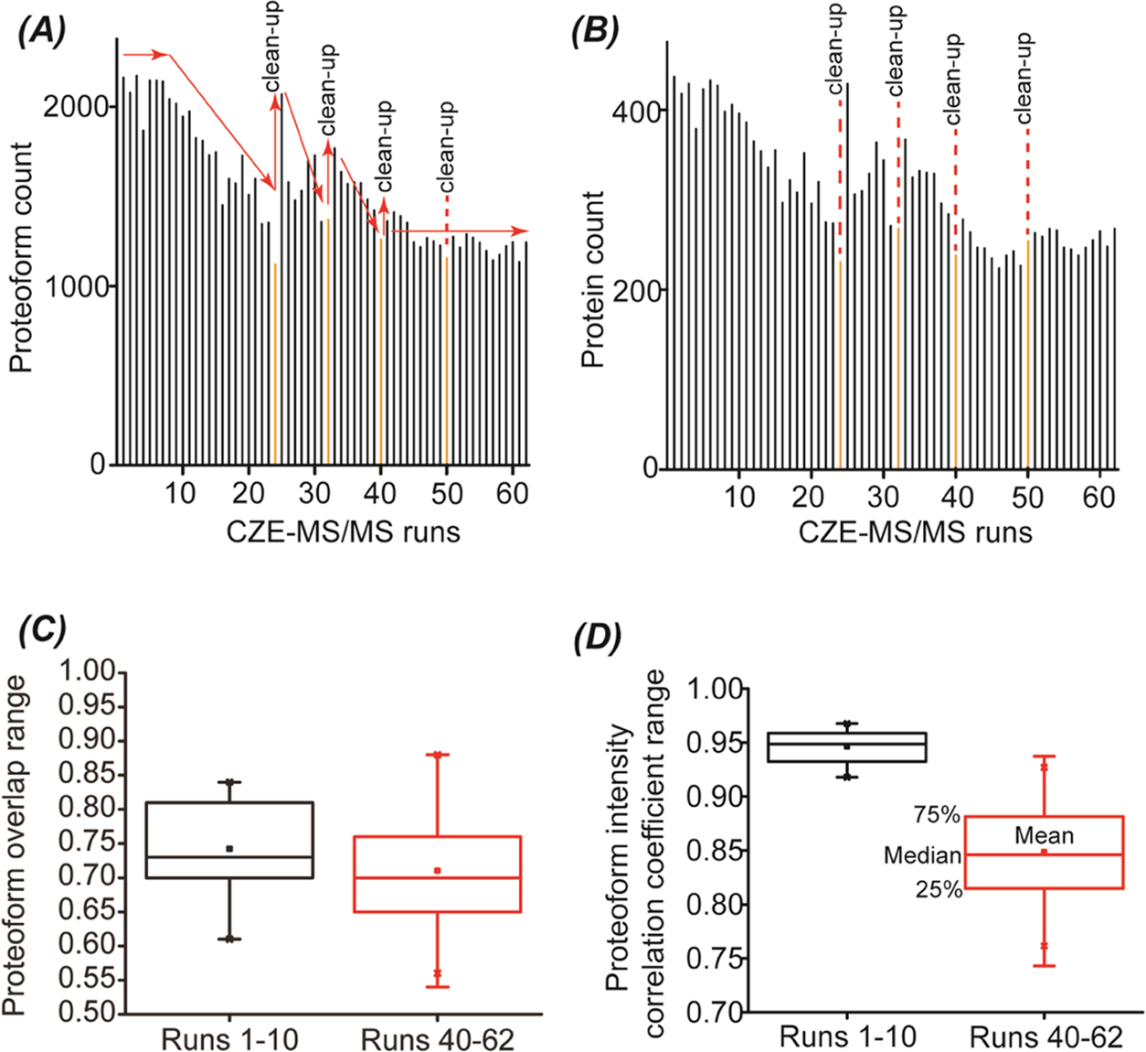
Summary of the identified proteoforms and proteins from 62 CZE-MS/MS runs. (A) The number of proteoform IDs was a function of the run number. (B) The number of protein IDs as a function of the run number. The trends of number of proteoform IDs and the time for capillary cleanup are marked. (C) Boxplots of pairwise proteoform overlaps for runs 1–10 and 40–62. (D) Boxplots of pairwise Pearson correlation coefficients of proteoform intensity for runs 1–10 and 40–62. Log 2 transformed proteoform intensities were used to generate the Pearson correlation coefficients.

CZE-MS/MS with a fresh LPA-coated capillary generated reproducible measurements of the yeast cell lysate, which is evidenced by the example electropherograms and the number of proteoform identifications from the first roughly 10 runs, Figures 2A and 3A. When we kept running the yeast cell lysate, we observed that the proteoform peaks were broadened gradually, and proteoform intensity decreased accordingly, Figure 2A. The peak width of one proteoform doubled in run 14 compared to that in run 1 and the proteoform intensity decreased by a factor of 2 roughly. For runs 16, 22, and 23, the peak width of the example proteoform tripled and the proteoform intensity is only 20% of that in run 1. The number of proteoform and protein IDs decreased obviously from run 10 to run 24, as shown in Figure 3A,3B.

We suspected that the phenomenon was due to proteoform adsorption onto the LPA polymer coating on the capillary’s inner wall. When more and more CZE-MS/MS runs are performed, proteoforms are gradually adsorbed onto the capillary wall. The adsorbed proteoforms can have significant impacts on the CZE separation. Proteoforms on the capillary inner wall are positively charged under the acidic BGE of 5% (v/v) acetic acid (pH 2.4), leading to a potential of generation of a low reversed electroosmotic flow (EOF) in the capillary. The reversed EOF slows down the migration of proteoforms in the capillary and increases the chance of peak broadening of proteoforms due to longitudinal diffusion and dispersion.^43^ The reversed EOF could also affect the performance of dynamic pH junction stacking because it could negatively impact the migration of hydrogen protons from the BGE vial to the separation capillary for sample zone titration.

Figure S1B shows an example electropherogram of the yeast cell lysate after more than 30 continuous CZE-MS/MS runs without capillary cleanup. Once we cleaned up the capillary inner wall using a procedure involving capillary flushing with 0.5% ammonium hydroxide, water, and the BGE, the separation profile and the number of proteoform IDs recovered back to nearly the original condition, Figure S1A,C. The data demonstrate that the cleaning up method can remove the adsorbed proteoforms efficiently.

After the first and second capillary cleanup, we observed the repeated phenomenon of the fresh capillary. The number of proteoform and protein IDs declined as the runs continued, Figure 3A,B. Interestingly, after the third cleanup, the capillary inner-wall condition became more stable, evidenced by the relatively more consistent numbers of proteoform and protein IDs (Figure 3A,B) as well as more reproducible proteoform separations (Figure 2B). Our data suggest that to achieve reproducible top-down MS measurements of a complex proteome sample by CZE-MS/MS, we can perform the experiment either using a fresh LPA-coated capillary (Phase I) or using an LPA-coated capillary after enough protein adsorption and sufficient capillary cleanup with 0.5% ammonium hydroxide (Phase II). The phase II condition can provide reproducible CZE-MS/MS measurements for more than 23 runs.

We further studied the pairwise overlap of identified proteoforms for phase I (runs 1–10) and phase II (runs 40–62) conditions, Figure 3C. The medians of proteoform overlap between any two CZE-MS/MS runs in phase I and phase II are both between 70 and 75%, which is comparable to CZE-MS/MS data in the literature.^44^ It documents that CZE-MS/MS under both conditions can repeatedly identify the same proteoforms from the yeast cell lysate. The small variations in the identified proteoforms are most likely due to the randomness of data-dependent acquisition (DDA).

To investigate the quantitative reproducibility of CZE-MS/MS in both phase I and II conditions, we studied the pairwise proteoform intensity correlation coefficients for runs 1–10 and 40–62, Figure 3D. Label-free quantification of proteoforms was performed by the TopDiff software.^41^ The intensities of overlapped proteoforms between any two runs were used to create the Pearson linear correlation and obtain the correlation coefficients. The median for the phase I runs is about 0.95, and the correlation coefficient has a narrow distribution, suggesting high quantitative reproducibility. The median for the phase II runs is about 0.85, indicating reasonable quantitative reproducibility. The much lower Pearson linear correlation coefficients in phase II runs than phase I runs are most likely due to drastically lower proteoform intensities in phase II runs, as shown in Figure 2A,B.

**Figure 4:**
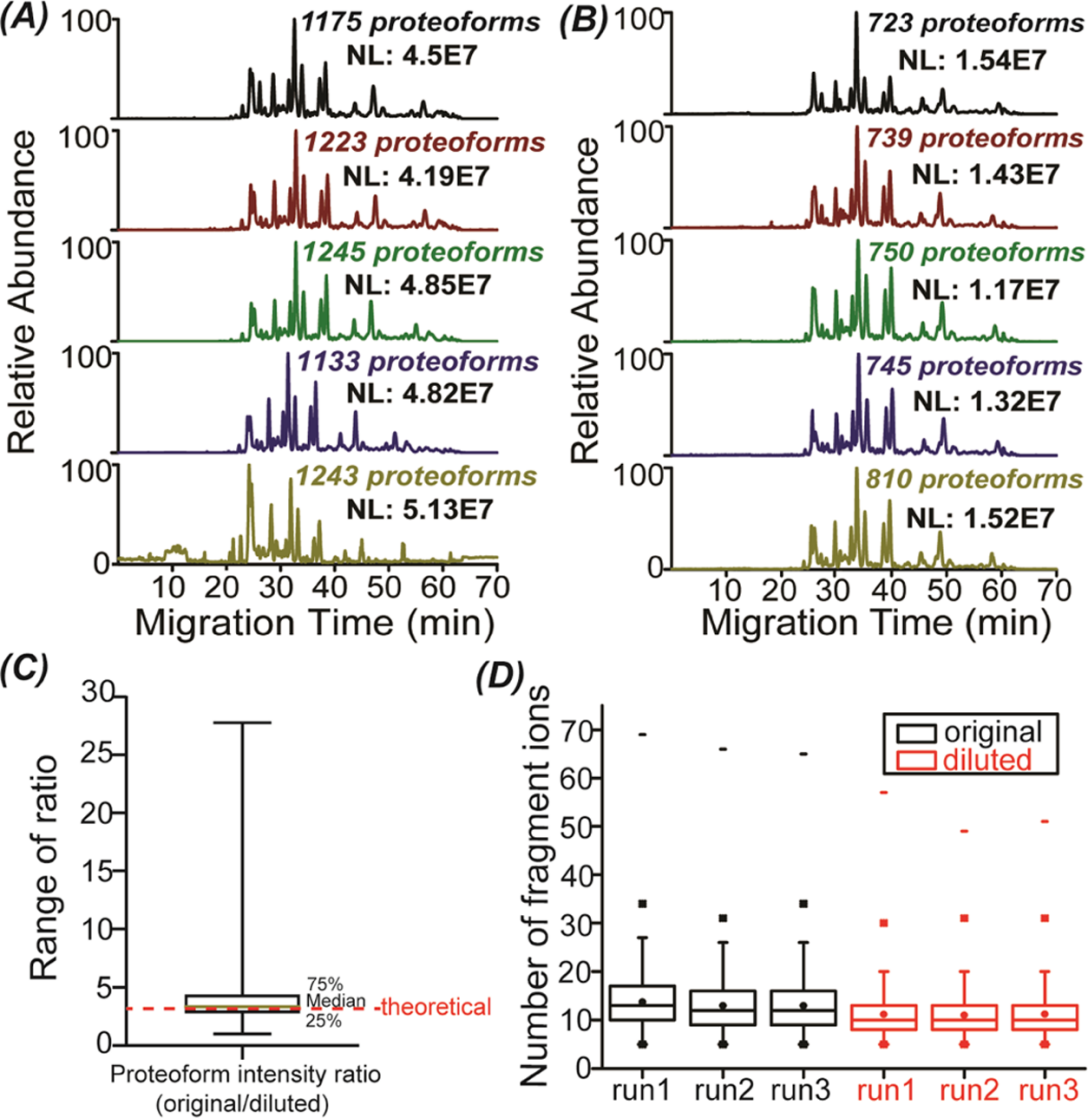
Comparisons of the original and diluted yeast cell lysate data from CZE-MS/MS analyses. (A) Base peak electropherograms of the original yeast cell lysate after CZE-MS/MS analyses in quintuplicate. (B) Base peak electropherograms of the 3 times diluted yeast cell lysate after CZE-MS/MS analyses in quintuplicate. (C) Boxplot of the intensity ratio of overlapped proteoforms between original and diluted yeast cell lysates. (D) Boxplots of the number of matched fragment ions of identified proteoforms from original and diluted yeast samples.

To further confirm the possibility of CZE-MS/MS in the phase II condition for accurate label-free quantification of proteoforms in a complex sample, after the 62 CZE-MS/MS runs of the yeast cell lysate, we performed CZE-MS/MS analyses of a 3 times diluted yeast cell lysate in quintuplicate, Figure 4. The CZE-MS/MS produced reproducible measurements of the original and diluted yeast cell lysates in terms of separation profiles, the number of proteoform IDs (1204 ± 49 for original vs 753 ± 33 for diluted, relative standard deviations (RSDs) as 4%, *n* = 5), and the normalized level (NL) intensities (RSDs: 8–9%, *n* = 5), Figure 4A,B. The average NL intensity of the diluted sample is about 3 times lower than that of the original sample (4.7E7 vs 1.4E7), which agrees well with the dilution factor of 3, demonstrating that the CZE-MS/MS in the phase II condition performs well for relative quantification of proteoforms. We further analyzed the distribution of proteoform intensity ratios between the original and diluted samples, Figure 4C. The median of the ratios is close to the theoretical ratio of 3. The number of matched fragment ions from the original sample is consistently higher than that from the diluted sample, most likely due to the much higher proteoform intensity, as shown in Figure 4D. Majority of the identified proteoforms have more than 10 matched fragment ions for the original and diluted samples, indicating reasonably high confidence of the proteoform IDs.

The results presented in this study are critically important for CZE-MS/MS for top-down proteomics of complex samples. First, the data document that CZE-MS/MS using one LPA-coated capillary can produce high-quality top-down proteomics data of a complex proteome sample for at least 78 h (67 runs and 70 min per run), indicating the high robustness of the system. Second, the study provides rich experimental data that can be extremely useful for pursuing a better understanding of CZE-MS for proteoform separation and characterization. Third, the results demonstrate that CZE-MS/MS with an appropriate operational procedure (i.e., capillary cleanup) can generate highly reproducible separation and identification of proteoforms in a complex sample across dozens of runs. The CZE-MS/MS is ready for some important biological applications to discover potentially critical proteoforms in biological processes and diseases in a quantitative manner. Fourth, the data also highlight some potential challenges of CZE-MS/MS for large-scale top-down proteomics studies in the next step and point out some important directions to work on. For example, we need to make more effort to create more consistent capillary inner-wall chemistry during CZE-MS/MS runs, which will eventually make CZE-MS/MS a powerful and highly reproducible technique for large-scale top-down proteomics studies.

### Correlation of Experimental and Predicted Electrophoretic Mobility of Proteoforms under Different CZE-MS/MS Conditions

We have shown that the electrophoretic mobility (μ_ef_) of proteoforms in CZE can be predicted well using a simple semiempirical model.^21,45^ Proteoforms’ experimental and predicted μ_ef_ have high linear correlation coefficients. This feature is critically useful for validating the proteoform IDs and PTMs (i.e., phosphorylation). Here, we have multiple different CZE-MS/MS conditions, phase I (runs 1–10), phase II (runs 40–62), and transition period between them (runs 11–39). We are asking how those CZE-MS/MS conditions influence the correlation of experimental and predicted μ_ef_ of proteoforms.

For the experimental μ_ef_ (cm^2^·kV^–1^·s^–1^), we used eq 1 for calculation.
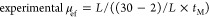
1where *L* is the capillary length in cm; *t*_M_ is the migration time in seconds; and 30 and 2 are the separation voltage and electrospray voltage in kilovolts, respectively.

For the predicted μ_ef_ (cm^2^·kV^–1^·s^–1^), we utilized eq 2.
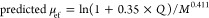
2where *M* and *Q* represent the molecular mass and charge number of each proteoform, respectively. We got the information on *M* directly from the database search results. We obtained *Q* by counting the number of lysine, arginine, and histidine amino acid residues in the proteoform sequence, and added 1 for the N-terminus.

**Figure 5:**
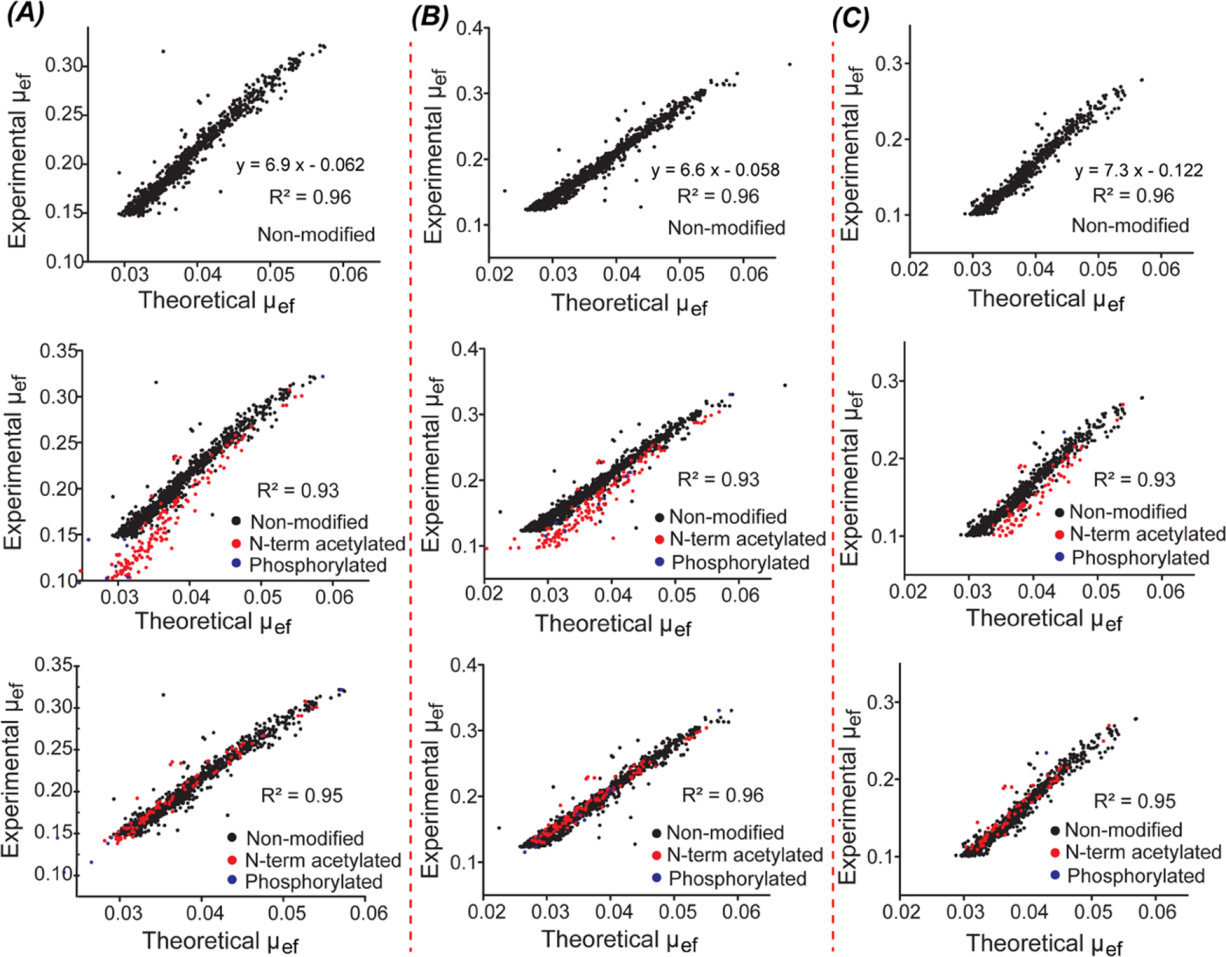
Linear correlations between predicted μ_ef_ and experimental μ_ef_ of proteoforms from the yeast cell lysate identified in CZE-MS/MS runs 5 (A), 25 (B), and 52 (C). The top figures show the correlations for proteoforms without any PTMs. The middle ones indicate the correlations for all proteoforms without PTMs and with N-terminal acetylation or phosphorylation. The charge *Q* of those proteoforms was not corrected. The bottom ones show the correlations for the same proteoforms as the middle ones but with charge *Q* correction. For example, for one N-terminal acetylation or phosphorylation, the *Q* was reduced by one.

We used only proteoforms containing no PTMs and those having N-terminal acetylation or phosphorylation for this study. As shown in Figure 5A–C (top panels), strong linear correlations between experimental and predicted μ_ef_ were observed for proteoforms without any PTMs (*R*^2^ = 0.96). As shown in the middle panels, when we consider the proteoforms with N-terminal acetylation or phosphorylation, those modified proteoforms fall off the main trend and have lower experimental μ_ef_ compared to the corresponding nonmodified proteoforms. The reduction of experimental μ_ef_ is due to the charge (*Q*) reduction by one from the N-terminal acetylation or phosphorylation, considering the acidic BGE of CZE (i.e., 5% acetic acid, pH 2.4). After reducing the estimated net charge *Q* by one for the μ_ef_ prediction, we achieved strong linear correlation coefficients (*R*^2^ = 0.95–0.96) for the nonmodified proteoforms and proteoforms having N-terminal acetylation or phosphorylation, Figure 5A–C (bottom panels). The results here suggest that the proteoforms identified in this study have high confidence because of the strong linear correlations between experimental and predicted μ_ef_. In addition, the data indicate that the different CZE-MS/MS conditions do not have a significant impact on the correlations between experimental and predicted μ_ef_. We realized that the experimental μ_ef_ of proteoforms become lower from run 5 (≥0.15, A) to run 52 (≥0.1, C), which is due to the much longer migration times of proteoforms in run 52 compared to run 5, Figure 2.

## Conclusions

For the first time, the long-term qualitative and quantitative reproducibility of CZE-MS/MS for a complex proteome sample was investigated. We revealed significant changes of proteoforms in migration time and intensity after about 10 CZE-MS/MS runs of the yeast cell lysate due to proteoform adsorption onto the capillary inner wall. We developed an efficient and simple capillary cleanup procedure via flushing the capillary with 0.5% NH_4_OH, water, and the separation buffer successively. The capillary cleanup protocol can remove the adsorbed proteoforms efficiently. After several rounds of capillary cleanup, the capillary inner wall chemistry became more consistent, producing reproducible proteoform separation and identification across dozens of CZE-MS/MS analyses of the yeast cell lysate. The results in this work highlight that CZE-MS/MS is robust enough to create high-quality top-down proteomics measurement of a complex sample across dozens of runs, for example, more than 60 runs of the yeast cell lysate. In addition, the measurement can be qualitatively and quantitatively reproducible across dozens of runs (i.e., at least 23 runs) under some specific conditions with an appropriate operational procedure (i.e., regular capillary cleanup). We expect that it is time to apply CZE-MS/MS-based top-down proteomics to broad biological applications.

We have some recommendations about using CZE-MS for quantitative top-down proteomics. For label-free quantification, we should not combine the Phase I and Phase II conditions because of the dramatic shifts in migration time, making the data alignment challenging across CZE-MS runs for relative quantification. If a small-scale label-free quantification is performed, for example, comparing two samples with only about 10 CZE-MS runs or fewer, then the Phase I condition will be ideal. If a large-scale study is needed, for example, comparing multiple samples with more than 20 CZE-MS runs, then the Phase II condition should be considered. Alternatively, stable isotopic labeling techniques (e.g., tandem mass tags^46^) can be employed. In this case, we may not need to worry about the Phase I or Phase II condition because the relative quantification is performed based on the data within the same CZE-MS runs.

## Supporting Information Available

Lists of identified proteoforms from all CZE-MS/MS runs (XLSX)
Electropherograms of a yeast cell lysate by CZE-MS/MS in three instances (PDF)
