# Supplementary material for: Pilot Evaluation of the Long-Term Reproducibility of Capillary Zone Electrophoresis–Tandem Mass Spectrometry for Top-Down Proteomics of a Complex Proteome Sample
Source: J Proteome Res. 2024 Feb 28;23(4):1399–407. doi: 10.1021/acs.jproteome.3c00872 (PMC11002928; doi:10.1021/acs.jproteome.3c00872)
Supplement: Supplementary file 2 — pr3c00872_si_002.pdf [file pr3c00872_si_002.pdf]

Supporting Information II

Pilot evaluation of long-term reproducibility of capillary zone electrophoresis-tandem mass spectrometry for top-down proteomics of a complex proteome sample

Seyed Amirhossein Sadeghi,[1] Wenrong Chen,[2] Qianyi Wang,[1] Fei Fang,[1] Xiaowen Liu,[3] Liangliang Sun[1,*]

[1]Department of Chemistry, Michigan State University, 578 S Shaw Lane, East Lansing, Michigan 48824 United States

[2]Department of BioHealth Informatics, Indiana University-Purdue University Indianapolis, 719 Indiana Avenue, Indianapolis, IN 46202, USA.

[3]Deming Department of Medicine, School of Medicine, Tulane University, 1441 Canal Street, New Orleans, LA 70112, USA.

* Corresponding Author. Email: lsun@chemistry.msu.edu Phone: 517-353-0498

Table of Contents

Supporting Information I:
The lists of identified proteoforms from all CZE-MS/MS runs **(XLSX)**

Supporting Information II:
Supplementary Figure 1. Example electropherograms of a yeast cell lysate by CZE-MS/MS in three instances

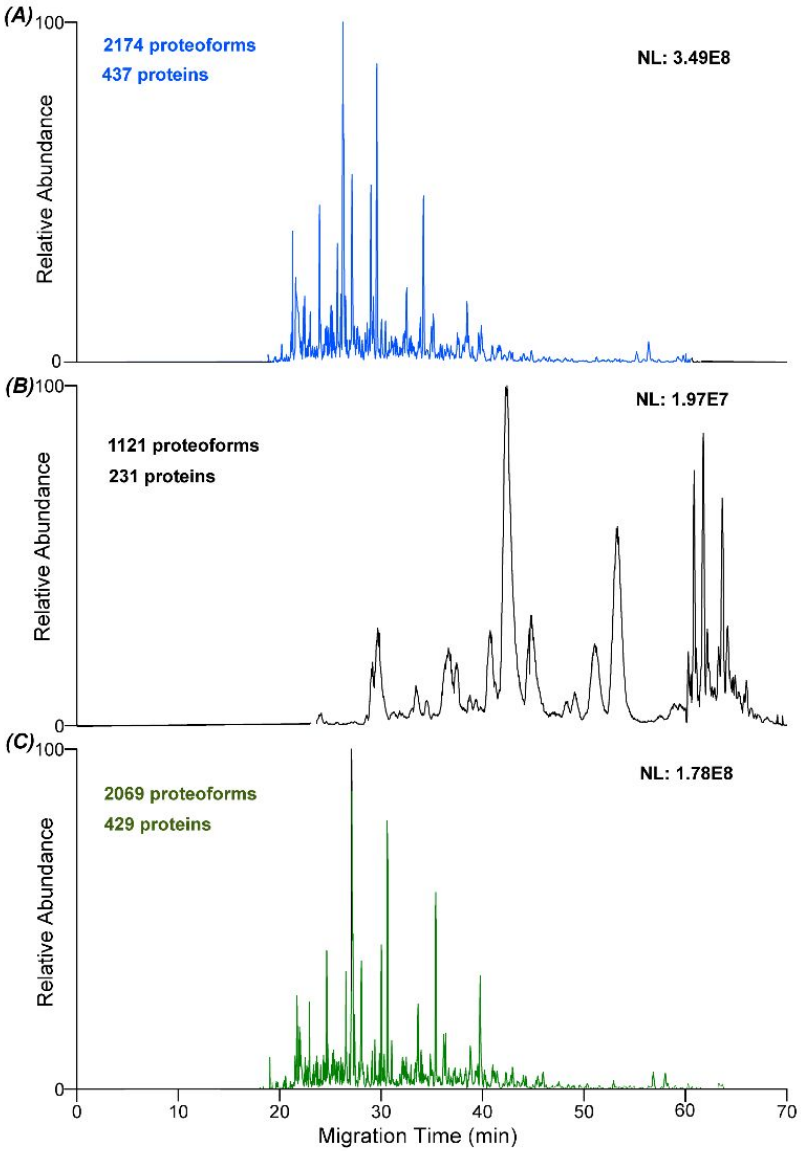

Figure S1. Example electropherograms of a yeast cell lysate by CZE-MS/MS in three instances. (A) a fresh separation capillary; (B) the capillary after 24 successive CZE-MS/MS runs; (C) the capillary after the first-time cleanup using 0.5% NH_4OH with the procedure in **Figure 1B**.
